# Determinants of preterm birth among mothers who gave birth in East Africa: systematic review and meta-analysis

**DOI:** 10.1186/s13052-020-0772-1

**Published:** 2020-01-28

**Authors:** Tariku Laelago, Tadele Yohannes, Gulima Tsige

**Affiliations:** 1 Department of Nursing, Wachemo University, Durame campus, Durame, Ethiopia; 2 College of Health Science and Medicine, Hawassa University, Hawassa, Ethiopia; 3 Hadiya Zone Health Department, Public Health Emergency Management, Hosanna, Ethiopia

**Keywords:** Preterm birth, Determinants, Systematic review, Meta-analysis

## Abstract

**Background:**

Preterm birth (PTB) can be caused by different factors. The factors can be classified into different categories: socio demographic, obstetric, reproductive health, medical, behavioral and nutritional related. The objective of this review was identifying determinants of PTB among mothers who gave birth in East African countries.

**Methods:**

We have searched the following electronic bibliographic databases: PubMed, Google scholar, Cochrane library, AJOL (African journal online). Cross sectional, case control and cohort study published in English were included. There was no restriction on publication period. Studies with no abstracts and or full texts, editorials, and qualitative in design were excluded. Funnel plot was used to check publication bias. I-squared statistic was used to check heterogeneity. Pooled analysis was done by using fixed and random effect model. The Joanna Briggs Critical Appraisal Tools for review and meta-analysis was used to check the study quality.

**Results:**

A total of 58 studies with 134,801 participants were used to identify determinants of PTB. On pooled analysis, PTB was associated with age < 20 years (AOR 1.76, 95% CI: 1.33–2.32), birth interval less than 24 months (AOR 2.03, 95% CI 1.57–2.62), multiple pregnancy (AOR 3.44,95% CI: 3.02–3.91), < 4 antenatal care (ANC) visits (AOR 5.52, 95% CI: 4.32–7.05), and absence of ANC (AOR 5.77, 95% CI: 4.27–7.79). Other determinants of PTB included: Antepartum hemorrhage (APH) (AOR 4.90, 95% CI: 3.48–6.89), pregnancy induced hypertension (PIH) (AOR 3.10, 95% CI: 2.34–4.09), premature rupture of membrane (PROM) (AOR 5.90, 95% CI: 4.39–7.93), history of PTB (AOR 3.45, 95% CI: 2.72–4.38), and history of still birth/abortion (AOR 3.93, 95% CI: 2.70–5.70). Furthermore, Anemia (AOR 4.58, 95% CI: 2.63–7.96), HIV infection (AOR 2.59, 95% CI: 1.84–3.66), urinary tract infection (UTI) (AOR 5.27, 95% CI: 2.98–9.31), presence of vaginal discharge (AOR 5.33, 95% CI: 3.19–8.92), and malaria (AOR 3.08, 95% CI: 2.32–4.10) were significantly associated with PTB.

**Conclusions:**

There are many determinants of PTB in East Africa. This review could provide policy makers, clinicians, and program officers to design intervention on preventing occurrence of PTB.

## Background

Preterm birth (PTB) is birth occurs between 20 weeks of pregnancy and 37 weeks of pregnancy. It is a concern because babies who are born too early may not be fully developed. They may be born with serious health problem. Some problems like cerebral palsy, can last a life time. Other problems like learning disabilities may appear later in childhood or even adulthood [1].

Each year 15 million babies in the world, more than one in 10 births is born too early. More than 1 million of those babies die shortly after birth; countless other suffer some type of life long physical, neurological or educational disabilities often at great cost to families and society [2]. The survival chances of the 15 million babies born preterm each year vary dramatically depending on where they are born. South Asia and sub-Saharan Africa account for half the world’s births, more than 60% of the world’s preterm babies and over 80% of the world’s 1.1 million deaths due to PTB complications. Around half of these babies are born at home. Even for those born in a health clinic or hospital, essential newborn care is often lacking. The risk of a neonatal death due to complications of PTB is at least 12 times higher for an African baby than for a European baby. Yet, more than three-quarters of PTB could be saved with feasible, cost-effective care, and further reductions are possible through intensive neonatal care [3].

PTB has multiple causes; therefore, solutions will not come through a single discovery but rather from an array of discoveries addressing multiple biological, clinical, and socio-behavioral risk factors. Age of mother [4], household income [5], educational status of mother [4, 6], place of residence and employment status [7] were associated with PTB. Many studies in different settings of the world revealed the contributing factors of PTB as physical activity [8], maternal cardiovascular disease [9], delivering by previous cesarean section [10], had history of miscarriage [11], and history of PTB [11–13]. The contributing factors for PTB also include pregnancy interval [14], body mass Indexes(BMII) [11], antenatal care(ANC) [12, 15, 16], multiple pregnancy [17], antepartum hemorrhage(APH) [15],urinary tract infections(UTI) [11], premature rupture of membrane(PROM) [15, 18], and pregnancy induced hypertension(PIH) [17, 19]. Moreover, marital status [12], polyhydramnios or oligohydramnios and genitourinary infections [20], periodontal disease [11], ascending infection (bacteriuria) [21] and exposure to intimate partner violence (IPV) [15, 22] are included in contributing factors of PTB.

So far different researches are done and published on determinants of PTB among mothers who gave birth in East Africa countries. However, the results of the studies were inconsistent, factors that had direct association in some studies may be inversely associated or had no association in other studies and vise-versa. Moreover, to the best of our knowledge, there is no pooled data on determinants of PTB in East Africa. Hence, this systematic review and meta-analysis was conducted to identify determinants of PTB among mothers who gave birth in East Africa countries. This will help to make conclusions based on best available scientific evidence. Moreover, the result of this review could support policy makers, clinicians, and programmers to design intervention on preventing PTB.

## Methods and materials

### Reporting

The report was written by using Preferred Reporting Items for Systematic Reviews and Meta-Analyses (PRISMA)) guideline [23]. This review was registered in PROSPERO database (PROSPERO 2019: CRD42019127645).

### Inclusion and exclusion criteria

Cross sectional, case control and cohort studies done in East African countries were included in this study. East African countries include (Sudan, South Sudan, Kenya, Uganda, Djibouti, Eritrea, Ethiopia, Somalia, Tanzania, Rwanda, Burundi, Comoros, Mauritius, Seychelles, Mozambique, Madagascar, Zambia, Malawi, Zimbabwe, Reunion, Mayotte) [24]. Studies reported the determinants of PTB and published in English were incorporated. There was no restriction on publication period. Studies with no abstracts and or full texts, editorials, and qualitative studies were excluded.

### Searching strategy and information sources

We have searched the following electronic databases: PubMed, Google scholar, Cochrane library, AJOL (African journal online). Furthermore, we have searched bibliographies and contacted authors. There was no restriction on publication period. To conduct a search of the literature databases, we have used Boolean Logic, connectors “AND”, “OR” in combinations [25]. The search strategy for PubMed database was done as following: preterm OR “preterm birth**”** OR “premature birth”(MeSH terms) AND birth OR parturition OR newborn(MeSH terms) OR infant OR child AND Sudan OR South Sudan OR Kenya OR Uganda OR Djibouti OR Eretria OR Ethiopia OR Somalia OR Tanzania OR Rwanda OR Burundi OR Comoros OR Mauritius OR Seychelles OR Mozambique OR Madagascar OR Zambia OR Malawi OR Zimbabwe OR Reunion OR Mayotte. The search algorithm for other database was done by modifying search strategy used for PubMed.

### Study selection

Studies retrieved from database were exported to reference note manager, endnote version 7 to remove duplicate studies. Title and abstract was screened by two reviewers. The full text of these potentially eligible studies was retrieved and independently assessed for eligibility by two review team members. Disagreement between them over the eligibility of particular studies was resolved through discussion with a third reviewer.

### Risk of bias in individual studies

To evaluate the quality of the papers, the Joanna Briggs Critical Appraisal Tools for review and meta-analysis was used [26]. Two independent reviewers assessed the quality of the study. Differences was reconciled by a third reviewer.

The following items was used to appraise case control studies:(1) comparable groups, (2) cases and controls matched, (3) the same criteria used for identification, (4) exposure measured in a standard, valid and reliable way, (5) exposure measured in the same way for cases and controls, (6) confounding factors identified, (7) strategies to deal with confounding factors, (8) outcomes assessed in a standard, valid and reliable way for cases and controls, (9) The exposure period of interest long enough, and (10) appropriate statistical analysis. Cohort studies were appraised by using the following items:(1) the two groups similar and recruited from the same population, (2) the exposures measured similarly to assign people to both exposed and unexposed groups, (3) the exposure measured in a valid and reliable way,(4)confounding factors identified, (5) strategies to deal with confounding factors, (6) participants free of the outcome at the start of the study, (7) the outcomes measured in a valid and reliable way, (8) follow up time reported and sufficient to be long enough for outcomes to occur, (9) follow up complete, and if not, were the reasons to loss to follow up described and explored, (10) strategies to address incomplete follow up, and (11) appropriate statistical analysis. For cross sectional studies the following items were used to appraise the quality: (1) criteria for inclusion, (2) study subjects and the setting described, (3) exposure measured in a valid and reliable way, (4) standard criteria used for measurement, (5) confounding factors identified, (6) strategies to appropriate statistical analysis deal with confounding factors, (7) outcomes measured in a valid and reliable way, and (8) appropriate statistical analysis.

Studies scored 50% and above in the quality assessment indictors were considered as low risk and included in the analysis.

### Data collection process

Two independent reviewers extracted data by using structured data extraction form. The name of the first author and year, country, study design, sample size, determinants of PTB, AOR (95% CI), events and total in experimental and control groups were extracted. Whenever variations of extracted data observed, the phase was repeated.

### Outcome measurement

PTB was considered, when newborn born < 37 weeks [27].

### Data analysis

To identify determinants of PTB, the analyses were divided in to six parts: Socio economic and demographic factors, reproductive health (RH), obstetric factors, medical condition, nutrition and behavioral factors. The Meta-analysis was done by using RevMan 5.3 software [28]. Heterogeneity of the studies was done by I-squared statistic (I^2^). A values of 25, 50, and 75% represented low, moderate, and high I^2^, respectively [29]. In this study I-squared value less than 50% was considered to interpret the combined effect size. Publication bias was checked by funnel plot. As the studies included in each outcome was less than 10, funnel plot was not presented [30]. Sensitivity analysis could investigate whether any indication of bias (such as different sizes of estimates from studies with individual participant data and from those without, or evidence of funnel plot asymmetry) remains when studies with individual participant data are standardized to match those lacking individual participant data [31]. We have conducted sensitivity analysis to see the effects of a single study on determinants of PTB. For small number of studies, it may be impossible to estimate the between studies variance with any precision. Therefore, we used fixed effect model for less than five studies and random effect model for five and above studies [32]. Pooled analysis was done using mantel-haenszel (M-H) statistical methods and effect measure was computed by odds ratio by using fixed and random effect model [30].

## Results

### Study characteristics

**Fig. 1:**
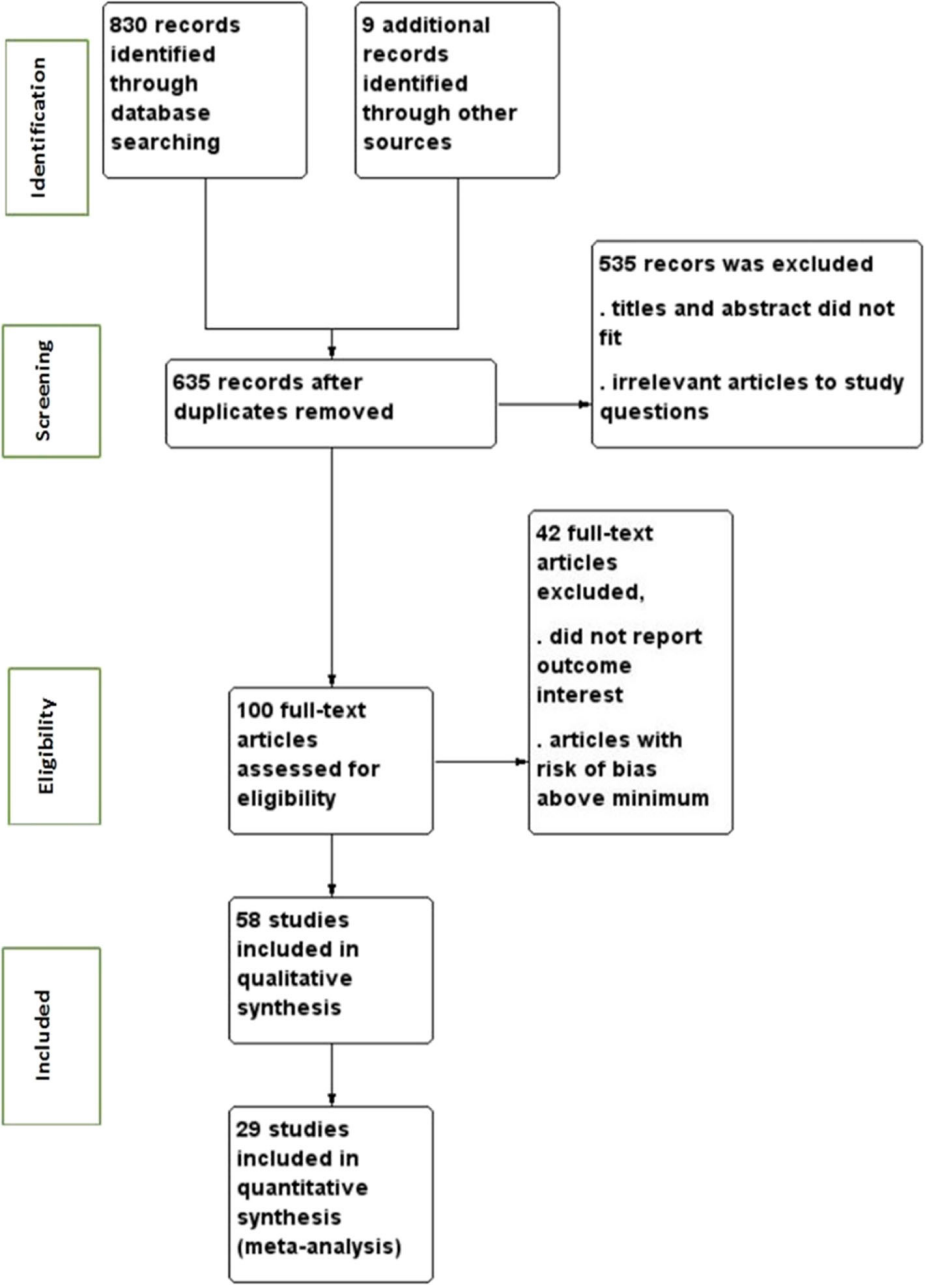
PRISMA study flow diagram showing data collection process

The search strategy retrieved 839 studies from databases and other sources. After duplication removed, 635 studies remained. Full text review was conducted for 100 studies and 58 studies with sample size of 134,801 participants were included to assess determinants of preterm birth (Fig. 1). The studies were included from 11 East African countries. Nineteen studies were from Tanzania [33–51], 11 from Ethiopia [52–63], 6 from Kenya [5, 54, 64–67], 7 from Malawi [4, 6, 68–72], 3 from Sudan [10, 73, 74], 3 from Zambia [75–77],3 from Zimbabwe [78–80], 2 from Uganda [7, 81], 2 from Mozambique [82, 83], 1 from Rwanda [84], and 1 from Madagascar [85]. Twenty five studies were done by cohort study design [33, 35, 36, 39, 41, 43, 45–50, 59, 61, 65–68, 70, 75, 77, 80, 83–85], 9 studies by case control study design [7, 10, 37, 38, 53, 55, 57, 73, 79] and 24 studies by cross sectional study design [4–6, 34, 40, 42, 44, 51, 52, 54, 56, 58, 60, 62, 64, 69, 71, 72, 74, 76, 78, 81, 82] (Table 1).

**Table 1:** Characteristics of studies included to study determinants of PTBS in East African countries

Author/year	Country	Study design	Sample size	Quality status
Abaraya et al. 2016 [57]	Ethiopia	Unmatched case control	656	Low risk
Abrams et al.2004 [72]	Malawi	Cross sectional	572	Low risk
Adam et al.2010 [74]	Sudan	Cross sectional	1200	Low risk
Adane et al. 2014 [60]	Ethiopia	Cross sectional	481	Low risk
Aidoo et al.2001 [67]	Kenya	Retrospective cohort	1077	Low risk
Alhaj et al.2010 [10]	Sudan	Case control	293	Low risk
Alson et al.2010 [85]	Madagascar	Cohort	206	Low risk
Arnaldo et al. *2018* [82]	Mozambique	Cross sectional	1038	Low risk
Ayebare E et al. 2018 [7]	Uganda	Case control	296	Low risk
Ayisi et al.2003 [66]	Kenya	Cohort	5168	Low risk
Berhanie et al. 2019 [53]	Ethiopia	Unmatched case control	954	Low risk
Chagomerana et al.2017 [68]	Malawi	Retrospective cohort	3074	Low risk
Chico et al.2017 [75]	Zambia	Prospective cohort	1086	Low risk
Cole et al.2001 [50]	Tanzania	Prospective cohort	1078	Low risk
Deborah Watson-Jones et al.2007 [48]	Tanzania	Prospective cohort	1688	Low risk
Deressa, et al. 2018 [56]	Ethiopia	Cross sectional	384	Low risk
Feresu et al.2004 [79]	Zimbabwe	Case control	3103	Low risk
Feresu et al.2004 [80]	Zimbabwe	Retrospective cohort	17,174	Low risk
Gebreslasie K 2016 [62]	Ethiopia	Cross sectional	540	Low risk
*Gesase* et al.*2018* [34]	Tanzania	Cross sectional	1117	Low risk
Habib et al.2008 [47]	Tanzania:	Cohort	16,762	Low risk
Kalanda et al.2006 [71]	Malawi	Cross sectional	4104	Low risk
Karki S 2016 [5]	Kenya	Cross sectional	691	Low risk
Kebede et al.*2013* [61]	Ethiopia	Retrospective cohort	416	Low risk
Kumwenda et al.2017 [76]	Zambia	Comparative cross sectional	200	Low risk
Laelago et al.2017 [58]	Ethiopia	Cross sectional	195	Low risk
Lepory et al.1998 [84]	Rwanda	Prospective cohort	1233	Low risk
Li et al.2016 [41]	Tanzania	Prospective cohort	3314	Low risk
Mace et al.2015 [77]	Zambia	Retrospective cohort	435	Low risk
Mahand et al.2013 [46]	Tanzania	Cohort	3359	Low risk
Mahande et al.2016 [39]	Tanzania	Retrospective cohort	17,030	Low risk
Mahande et al.2016 [39]	Tanzania	Retrospective cross sectional	30,797	Low risk
Mahande, et al. 2017 [37]	Tanzania	Matched case control	100	Low risk
Mahapula et al. 2016 [38]	Tanzania	Case control	754	Low risk
McDonald CR, et al.2015 [43]	Tanzanian	Cohort	1054	Low risk
Mekonen et al.*2019* [52]	Ethiopia	Cross sectional	575	Low risk
Menendez et al.2000 [51]	Tanzania.	Cross sectional	1225	Low risk
Mochache KM/2016 [65]	Kenya	Prospective cohort	292	Low risk
Mosha et al.2015 [36]	Tanzania.	Cohort study	2167	Low risk
Mosha et al.2016 [36]	Tanzania	Prospective cohort	7634	Low risk
Mpogoro et al.2014 [44]	Tanzania	Cross sectional	431	Low risk
Muti et al.2015 [78]	Zimbabwe	Cross sectional	287	Low risk
Muti et al.2015 [78]	Zimbabwe	Cross sectional	287	Low risk
Ndeserua [42] et al.2015	Tanzania	Cross sectional	350	Low risk
Okube et al. 2017 [64]	Kenya	Cross sectional	184	Low risk
Osman et al.2001 [83]	Mozambique	Prospective cohort	908	Low risk
Rempis’ et al. 2017	Uganda	Cross-sectional	412	Low risk
Sharif et al2017 [73]	Sudan	Case control	112	Low risk
Sigalla et al. 2017 [35]	Tanzania	Prospective cohort	1133	Low risk
Stephen et al.2018 [33]	Tanzania	Cohort	529	Low risk
Sullivan et al.1999 [6]	Malawi	Cross sectional	178	Low risk
Taha et al.2012 [4]	Malawi	Cross sectional	8874	Low risk
Teklay et al.*2018* [55]	Ethiopia	Un-matched retrospective case–control	264	Low risk
Turner et al.2013 [70]	Malawi	Cohort	809	Low risk
Van den Broek et al.2014 [69]	Malawi	Cross sectional	2149	Low risk
Wagura et al. 2018 [54]	Kenya	Cross sectional	322	Low risk
Watson-Jones et al.2002 [49]	Tanzania	Retrospective cohort	380	Low risk
Zerfu et al.2016 [59]	Ethiopia	prospective cohort	432	Low risk

### Risk of bias within studies

Joanna Briggs Critical Appraisal Tools for review and meta-analysis for case control studies, cross sectional studies and cohort studies were used. We included studies that had low risk (Table 1).

### Sensitivity analyses

On history of still birth, Feresu et al. 2004 [79], PIH, Deresa et al. 2018 and Abaraya et al.2018 [56, 57] and PROM, Ayebare et al. 2018 [7] had shown impact. On anemia, Abaraya et al.2018 and Remsi et al.2107 [57, 81] had revealed impact. On multiple pregnancy, Teklya et al. 2018 and Mahapula et al.2016 studies [38, 55] had brought out effects. Likewise, on ANC < 4 visits, Teklya et al. 2018 [55] and absence of ANC visits, Bekele et al. 2016 and Feresu 2004 et al. [63, 79] disclosed effects. Moreover, on HIV positive, Coley et al.2001 and Deressa et al.2018 [50, 56], and shorter pregnancy interval, Mahande et al. 2016 [39] showed effects. Besides, on UTI, Mahapula et al.2016 [38], malaria, Rempis et al.2017 [81], and obstetric complication Mekonen et al. 2018 [52] had shown impact. The abovementioned studies were become out of pooled analysis.

### Factors associated with PTB

#### Socio economic and demographic factors

**Fig. 2:**
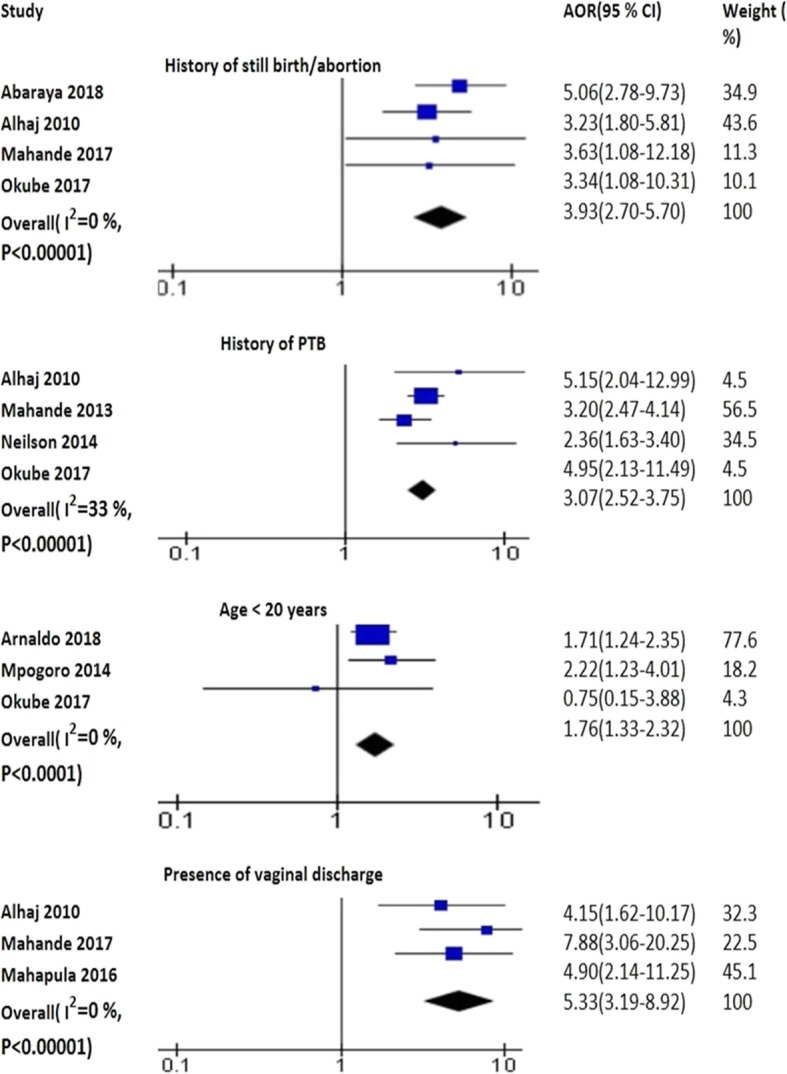
The pooled effects of age, vaginal discharge, history of PTB and still birth on PTB

The pooled analysis of three studies identified that age less than 20 years increased the probability PTB (AOR 1.76; 95% CI: 1.33–2.32) [44, 64, 82] (Fig. 2). Age of sexual debut at 16–18 years (AOR 2.17; 95% CI: 1.2–3.8) and 18–30 years (AOR 1.99; 95% CI: 1.1–3.6) [48] was associated with PTB. Younger maternal age was protective for PTB (AOR 0.98; 95% CI: 0.96–1.00) [4].

Household income ≤ US$ 97.85 (AOR 2.7; 95% CI: 1.4–5.0) [5] and low income < 600 birr (AOR 2.6; 95% CI: 1.1, 6.6) [63] was significantly associated with PTB. Women with no education had higher probability to have PTB (AOR 3.50; 95% CI: 1.58–7.77) [6]. Higher maternal education was protective against PTB (AOR 0.70; 95% CI: 0.51–0.95) [4]. Delivery in rainy season increased the risk of PTB (AOR 3.93; 95% CI: 1.75–8.79) [6]. Maternal weight less than 50 kg (AOR 5.1; 95% CI: 1.7–15.9) [72] and weight gain < 1 kg increased the probability of having PTB (AOR 2.64; 95% CI: 1.39–5.02) [83] whereas weight gain during pregnancy (AOR 0.89; 95% CI 0.82–0.97) reduced the odds of PTB [69]. Later birth year was associated with lower PTB risk (AOR 0.35, 95% CI: 0.19–0.70) [4]. Female gender (AOR 1.24; 95% CI: 1.04–1.48) [4] and being adolescents (AOR 2.60, 95% CI: 1.16–5.78) [76] increased risk of PTB. Dwelling in rural area (AOR = 6.56; 95% CI: 2.64–16.10) and being unemployed (AOR 0.36; 95% CI: 0.15–0.86) [7] were associated with PTB. MUAC of mother 17–28.5 cm (ARR 0.95; 95% CI: 0.92, 0.99) [79] and less than 24 cm (AOR 2.6; 95% CI 1.1–6.1) [52] was significantly associated with PTB. Increasing in BMI (AOR 0.91, 95%, CI 0.85–0.97) [69] reduced the probability of PTB whereas BMI < 18.499 increased the risk of PTB (AOR 4.52, 95% CI: 2.39–9.27) [61]. Being primigravida was positively associated with PTB (AOR 2.3; 95% CI: (1.3–4.0) [71].

The effects of IPV on PTB were examined by three studies. Of three, two studies found positive association between IPV and PTB [35, 53] and one found no association [58]. Women exposed to IPV (AOR 2.5; 95% CI: 2.19–2.96) and physical violence (AOR 5.3; 95% CI: 3.95–7.09) during pregnancy were more likely to experience PTB [53]. Furthermore, women exposed to physical IPV (AOR 2.9; 95% CI: 1.3–6.5) and women with previous adverse pregnancy outcomes (AOR 4.5; 95% CI: 1.5–13.7) were more likely to experience PTB [35].

#### Reproductive health factors

**Fig. 3:**
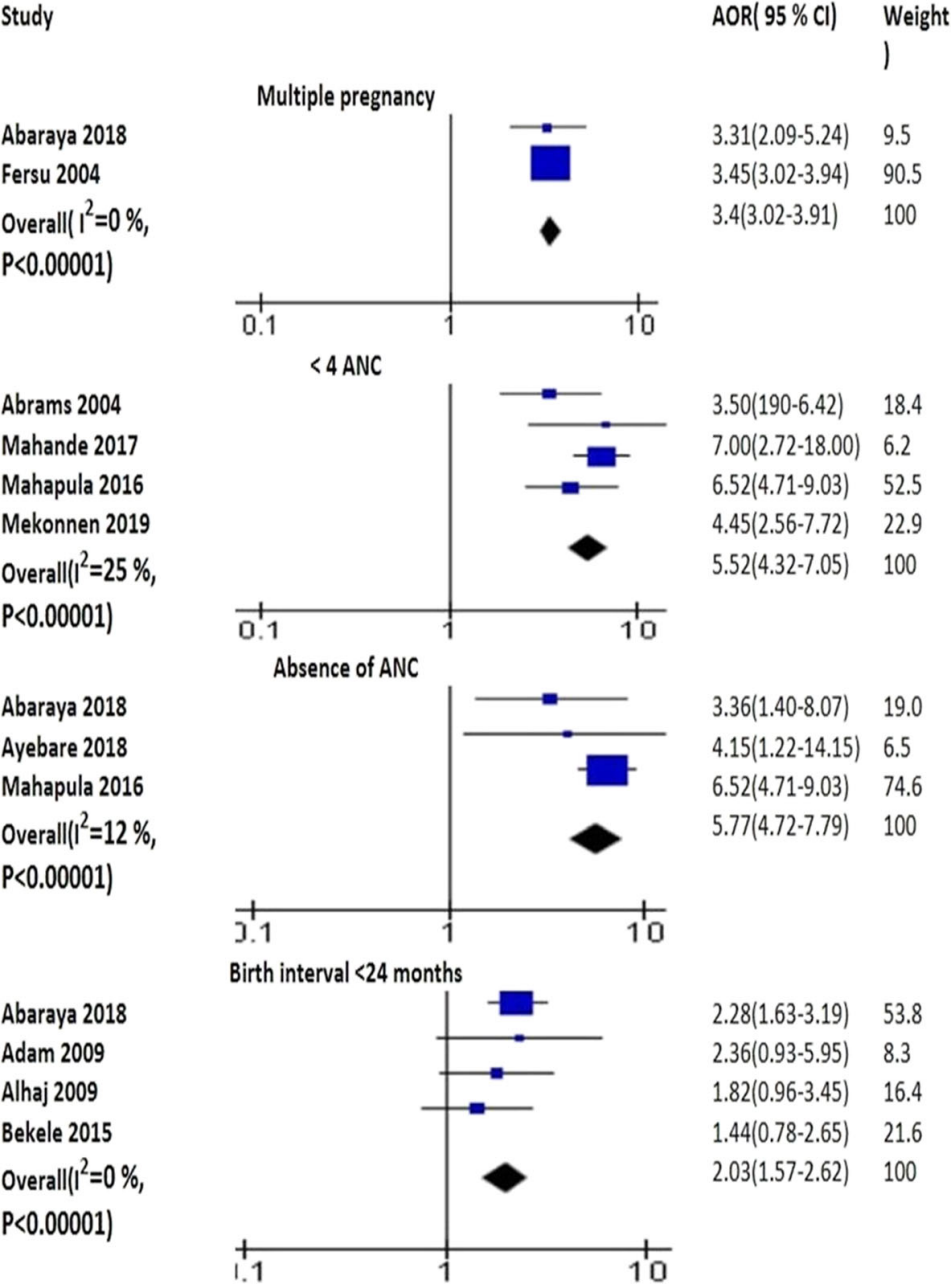
The pooled effects of multiple pregnancy, ANC and birth intervals on PTB

The pooled effects of four studies illustrated that birth interval less than 24 months was positively associated with PTB (AOR 2.03; 95% CI: 1.57–2.62) [10, 57, 63, 74]. The pooled analyses of four studies (AOR 5.52; 95% CI: 4.32–7.05) [37, 38, 52, 72] and three studies (AOR 5.77, 95% CI 4.27–7.79) [7, 38, 57] showed that ANC visits < 4 and absence of ANC increased the risk of PTB, respectively. The pooled effects of two studies identified that multiple pregnancy was positively associated with PTB (AOR 3.44; 95% CI: 3.02–3.91) [57, 79] (Fig. 3). Infants conceived after longer inter-pregnancy interval (≥60 months) had more chance to be PTB (AOR 1.13, 95% CI: 1.02–1.24) [39]. Mothers who had no PMTCT (prevention mother to child treatment) intervention had higher risk of having PTB [61]. Absence of public prenatal care (AOR 2.1; 95% CI: 1.1–4.1) [38] and ANC visits < 5 (AOR 2.2, 95% CI 1.3–3.7) [71] increased the risk of PTB.

#### Obstetric complications

**Fig. 4:**
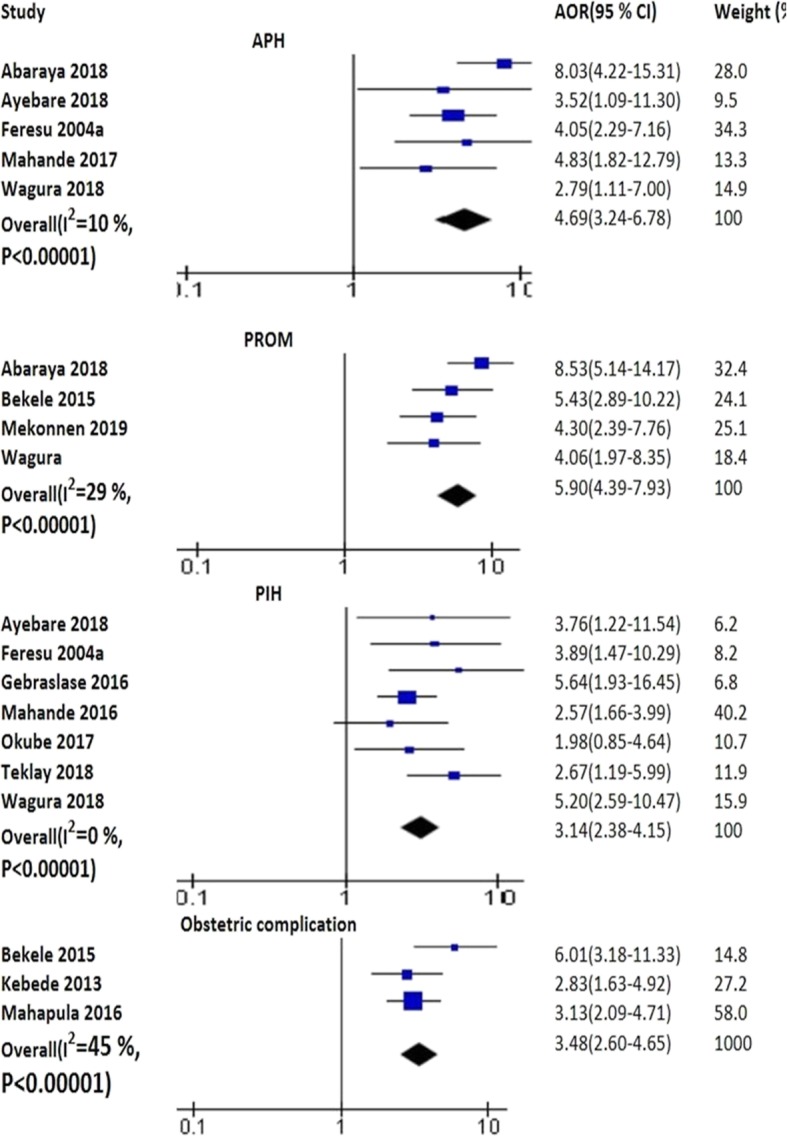
The pooled effects of APH, PROM, PIH and obstetric complications on PTB

The pooled analysis of five studies disclosed that APH increased the risk of PTB (AOR 4.90; 95% CI: 3.48–6.89) [7, 37, 54, 57, 79]. The pooled effects of seven studies revealed that PIH was significantly associated with PTB (AOR 3.10; 95% CI: 2.34–4.09) [7, 46, 54, 55, 62, 64, 79]. The pooled analysis of four studies pointed out an association of PROM with PTB (AOR 5.90; 95% CI: 4.39–7.93) [52, 54, 57, 63]. In addition, obstetric complications was associated with PTB as depicted by the pooled analysis of three studies (AOR 3.48; 95% CI: 2.60–4.65) [38, 61, 63] (Fig. 4).

Fertility treatment before this pregnancy (AOR 7.0; 95% CI: 1.90–27.26), uterine pain in the current pregnancy (AOR 5.0; 95% CI: 1.7–14.35) and regular menstrual bleeding (AOR 5.8 (2.3–14.86) increased odds of PTB [37]. Cervical incompetence (AOR 11.6, 95% CI: 1.1–121.5) and polyhydramnios (AOR 8.3; 95% CI: 1.7–40.2) were reported by mothers who gave PTB [38]. History of cesarean delivery (AOR 5.4, 95% CI: 1.7–17.3] [10], history of either PTB or small baby (AOR 3.1; 95% CI 1.1–8.4) [60], history of a previous PTB (AOR 2.13; 95% CI: 1.19–3.80) [61] raised the probability of PTB.

The presence of chorio-amnionitis (AOR 3.8; 95% CI: 1.3–10.8) [72] and placenta praevia was correlated with PTB (ARR 3.30, 95% CI: 1.34, 8.14) [79]. The pooled analysis of four studies displayed that history of still birth/abortion was positively associated with PTB (AOR 3.93; 95% CI: 2.70–5.70) [10, 37, 57, 64]. The pooled effects of four studies displayed that history of PTB increased the odds of PTB (AOR 3.45; 95% CI: 2.72–4.38) [10, 46, 64, 69] (Fig. 2). Fetal distress (AOR 4.0; 95% CI: 1.9, 8.2) and birth defects (AOR 3.20; 95% CI: 1.22–8.32) increased the odds of PTB [55]. Low birth weight (RR 2.9; 95% CI: 2.3–3.6) and perinatal death (RR 2.5; 95% CI: 1.9–3.5) raised the risk of PTB [46].

#### Medical conditions

**Fig. 5:**
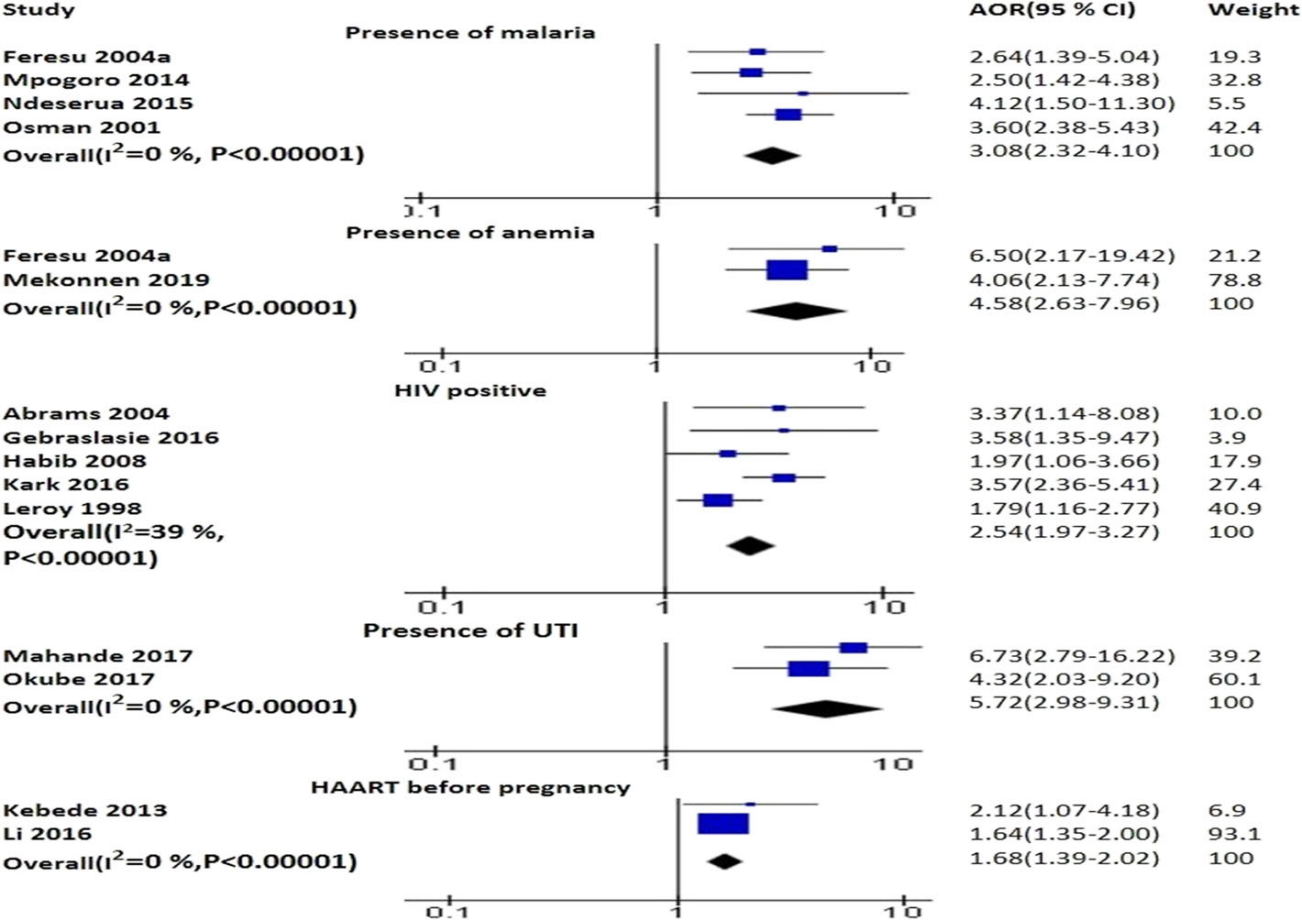
The pooled effects of malaria, anemia, HIV and HAART exposure on PTB

The pooled effects of two studies displayed that anemia was positively associated with PTB (AOR 4.58; 95% CI: 2.63–7.96) [52, 79]. HIV infection (AOR 2.59; 95% CI: 1.84–3.66) [5, 47, 62, 72, 84] and mothers who had started HAART before pregnancy was associated with PTB as identified by the pooled analysis of studies (AOR 1.68; 95% CI: 1.39–2.02) [41, 61]. Presence of malaria was associated with PTB as evidenced by the pooled analysis of four studies (AOR 3.08; 95% CI: 2.32–4.10) [42, 44, 79, 83]. The pooled analysis of two studies identified that UTI was significantly associated with PTB (AOR 5.27; 95% CI: 2.98–9.31) [37, 64] (Fig. 5). Presence of vaginal discharge increased the risk PTB as demonstrated by the pooled effects of three studies (AOR 5.33: 95% CI: 3.19–8.92) [10, 37, 38] (Fig. 2).

Women with unknown HIV status had moderately increased risks of having PTB (ARR 1.40; 95% CI: 1.23–1.59) [47]. Compared with women infected with HIV alone, primigravida with dual infection of HIV and malaria had an increased risk of delivering a PTB (AOR 3.4; 95% CI: 1.8–6.4) [86]. Baseline maternal CD4 level below 200/mm3 was significantly associated with PTB (AOR 5.37; 95% CI: 1.86–15.49) [61]. Presence of cord blood parasitemia was positively correlated with PTB (AOR 3.34; 95% CI: 1.26–8.82) [6]. The presence of maternal malaria increased the probability of having PTB (AOR 3.19, 95% CI: 1.9–5.2) [48]. Untreated bacterial vaginosis increased the probability of PTB (AOR 2.95, 95% CI: 1.3–6.6) [48].

TNF2 *(*Tumor Necrosis Factor) homozygosity was associated with PTB when compared with *TNF1* homozygotes (RR 7.3, 95% CI: 2.85–18.9) and heterozygotes (RR 6.7, 95% CI: 2.0–23) [67]. Women with plasma levels of Chitinase-3-Like Protein-1(CHI3L1(AOR 2.82; 95% CI:1.56, 5.08)), C5a(AOR 1.94; 95% CI: 1.15–3.29), soluble Intercellular Adhesion Molecule-1(sICAM-1(AOR 1.91; 95% CI:1.12, 3.29), and Interleukin-18 Binding Protein(IL-18BP (AOR 2.60; 95% CI:1.47, 4.63) in the highest quartile had an increased risk of PTB compared with those in the lowest quartile. Women with Leptin (AOR 0.39; 95% CI: 0.20, 0.73) and Angiopoietin (Ang2 (AOR 0.48; 95% CI: 0.27, 0.85) in the highest quartile had a reduced risk of PTB compared with women in the lowest quartile [43].

Women with high-titer active syphilis were at the greatest risk of having PTB (ARR 6.1 (2.5–15.3) [49]. Presence of periodontal disease (AOR 2.32; 95%CI: 1.33–4.27) [34] and periodontitis (at least three sites from different teeth with clinical attachment loss greater than or equal to4 mm [85] was significantly associated with PTB. Maternal depression increased the probability of having PTB (ARR 4.13; 95% CI: 2.82–17.42) [65]. Hematocrit level < 33 (AOR 7.2; 95% CI: 3.1–16.8) [63] and presence of chronic illness (AOR 4.5; 95% CI: 2, 10.2) [63] were found to be significantly associated with PTB. Maternal parasitized red blood cells <=10(AOR 1.9; 95%CI: 1.1–3.4), > 10(AOR 3.2; 95% CI: 1.5–7.0) and perivillous fibrin deposition> 30 (AOR 2.1; 95% CI: 1.3–3.5) were associated with increased risk of premature delivery [51].

#### Nutritional factors

Women in the inadequate dietary diversity group had a higher risk for PTB (ARR 4.61; 95% CI: 2.31, 9.19) [59]. Median folic acid level 8.6 ng/mg (OR 0.64; 95% CI: 0.53–0.77) [73], higher calcium intake (RR 0.76; 95% CI: 0. 65–0.88) and dietary animal Fe (Iron) intake (RR 0.67; 95% CI: 0.51–0.90) reduced the probability of PTB [36].

#### Behavioral factors

Drank home brew during pregnancy reduced the probability of having PTB (ARR 0.75; 95% CI: 0.60, 0.93) [79]. Use of traditional medication in pregnancy (AOR 5.6; 95% CI: 2.1–14.87) [37] and quinine exposure in first trimester was associated with an increased risk of PTB (OR 2.6; 95% CI: 1.3–5.3) [45]. Women who did not take IPT (Intermittent preventive treatment) had higher chance to have PTB (AOR 21.0, 95% CI: 2.9–153.8) [72]. Use of at least 2 doses of SP (sulphadoxine-pyrimethamine) for IPT during pregnancy (AOR 0.1, 95% CI: 0.05–0.4) [42], two or more doses compared to 0–1 dose reduced preterm delivery (OR, 0.42; 95% CI: 0.27, 0.67) [75]. Among multigravida women, at least two or more doses of SP-IPTp SPIPTp (sulphadoxine-pyrimethamine for IPT remained significantly associated with protection from PTB (AOR 0.28, 95% CI: 0.13–0.60) [77]. Last SP time lapse ≤4 weeks reduced the PTB (AOR 0.38, 95% CI: 0.15, 0.97) [44].

#### Conceptual frame work

**Fig. 6:**
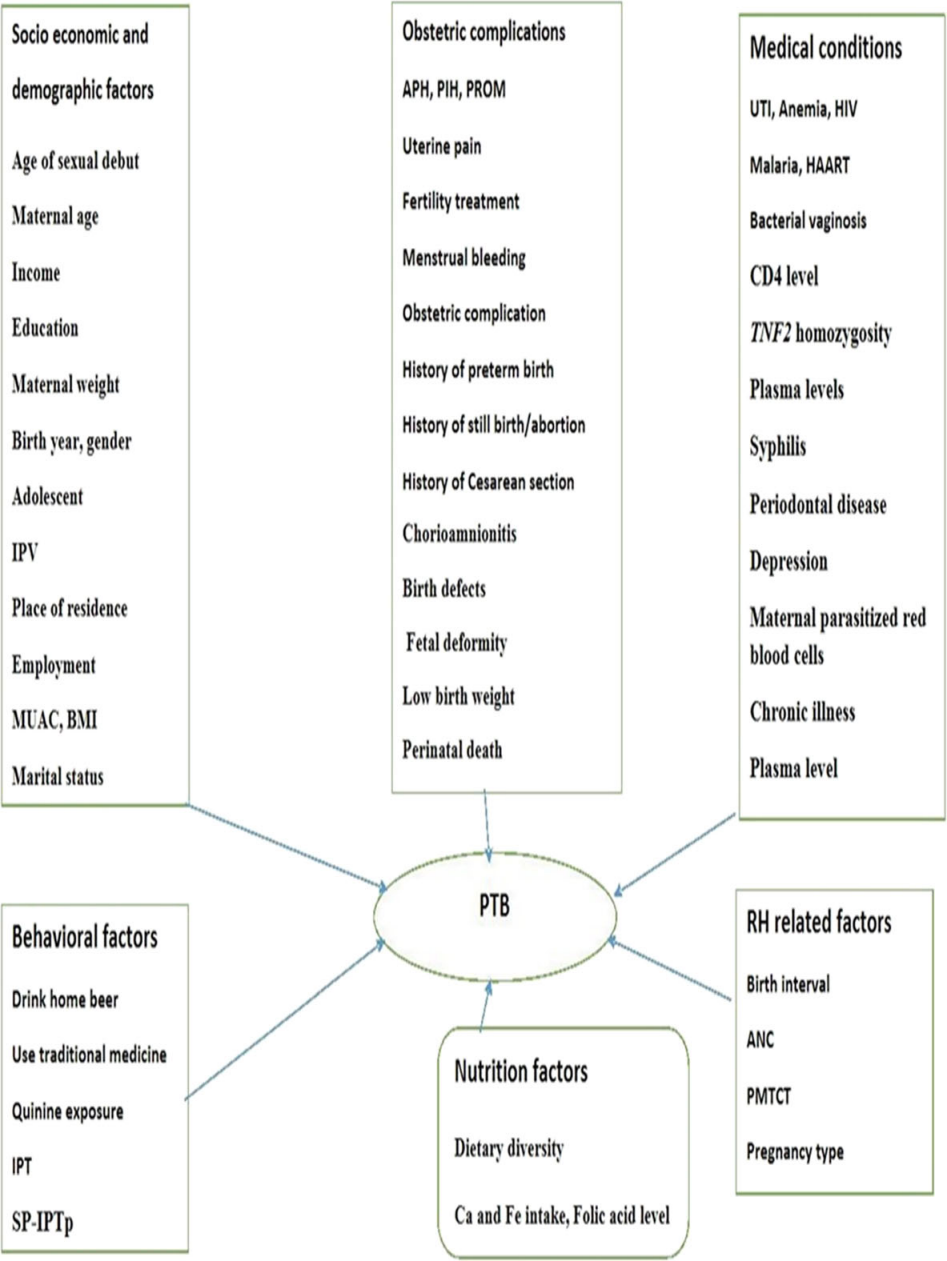
Conceptual framework showing determinants of PTB in East Africa

The conceptual framework of the study showing determinants of PTB in East Africa (Fig. 6).

## Discussion

The objective of this systematic review and meta-analysis was identifying determinants of PTB in East Africa. Age of women less than 20 years was correlated with PTB. This is comparable with other studies [22, 87]. The increased risk PTB in younger age can be linked to the fact that their reproductive organs are not yet fully developed.

The current study depicted that history of still birth/abortion was significantly associated with PTB. This is related with systematic review and meta-analysis study [11, 88]. History of PTB was significantly associated with PTB. This is in agreement with other studies [11–13, 19, 89]. The reason for this could be the likelihood of having PTB with the women with prior spontaneous labor as well as those with inducing PTB rising. PROM was significantly associated with PTB. This is similar with systematic review and meta-analysis study [18].

Shorter pregnancy interval was significantly associated with PTB. This is in line with studies done in Egypt and other places [11, 14, 90]. This may be because mothers do not have time to recover from the physical stress and nutritional burden of the pregnancy.

Women who attended ANC < 4 times had higher probability to have PTB. This finding is comparable with systematic review and meta-analysis study conducted in Iran [16]. Likewise, the absence of ANC was significantly associated with PTB. The reason for this might be when women had no chance to attend ANC, she cannot be informed of early identification of risk factors associated with PTB. Consequently, the probability of having PTB will increase.

Having multiple pregnancy increased the probability of PTB. It is in line with other studies [17, 91].This may be due to overstretching of uterus and deciding to complete pregnancy before term. Furthermore, it may be due to spontaneous labor or PROM.

Maternal UTI was associated with PTB. This is in agreement with other studies [11, 15, 19]. UTI can weaken the membranes of the amniotic sac around the baby. This could lead to PROM and preterm labor [92]. Presence of malaria increased the risk of PTB. This is in line with other studies [15]. Presence of anemia was associated with PTB. This is in agreement with other study [15]. Anemia can decrease blood flow to placenta and this can causes placental insufficiency, finally results in PTB. Presence of vaginal discharge during pregnancy increased the chance of having PTB. This is alike with other study [37].

Women who were already on HAART preconception had higher probability to have PTB. This is in line with other studies [93, 94]. HIV positive women had more probability to give PTB than HIV negative women. This is in agreement with study done in South Africa [95]. Presence of periodontal disease was significantly associated with PTB as depicted systematic reviews of studies. Study done in Egypt showed similar result [11]. This may be due to periodontal can results in an increase of pro-inflammatory molecules that can directly or indirectly lead to uterine contractions and cervical dilatation.

From determinants of PTB, PROM [12, 13, 15, 19, 96, 97], APH [13, 15, 19], ANC status [12, 13, 19], PIH [13, 19, 97], History of PTB [11, 13, 19], maternal age [13, 15, 97] multiple pregnancy [13, 15, 96] was prevalent in other African countries. The possible reason for this could be poor management of maternal health problems, limited access to health facilities and low health seeking behavior of the community. Birth interval less than 24 months, age less than 20 years, ANC status, and anemia could be more quickly modified.

The strength of this study is that this review seems to be the first done in East Africa, indicating the various determinants of PTB from 58 studies. This study has the following limitations. The search strategy was limited to studies published in English language, this can cause reporting bias. Data were not found for 10 East African countries, this can cause representativeness problems. Thus, further studies on determinants of PTB should be conducted in all East African countries by using standard WHO definition of PTB.

## Conclusions

There are many determinants of PTB in East Africa. The determinants can be categorized into socio economic and demographic factors, RH, obstetric complications, medical condition, and behavior related factors. This review could provide policy makers, clinicians, and program officers to design intervention on preventing the occurrence of PTB.

## Data Availability

All data generated or analyzed during this study are included in this published article.

## Abbreviations

ANC: Antenatal care
APH: Antepartum hemorrhage
ART: Anti-retroviral therapy
BMI: Body mass index
HAART: Highly active anti-retroviral therapy
HIV: Human immune deficiency virus
IPT: Intermittent preventive treatment
IPTp-SP: Intermittent preventive treatment of malaria in pregnancy
IPV: Intimate partner violence
MUAC: Mid-upper arm circumference
PIH: Pregnancy induced hypertension
PMTCT: Prevention mother to child transmission
PRISMA: Preferred Reporting Items for Systematic Reviews and Meta-Analyses
PROM: Premature rupture of membrane
PTB: Preterm birth
RH: Reproductive health
TNF: Tumor necrosis factor
UTI: Urinary tract infection
WHO: World health organization
